# Supplementary material for: Double-layer geodesic and gradient-index lenses
Source: Nat Commun. 2022 Apr 29;13:2354. doi: 10.1038/s41467-022-29587-9 (PMC9054803; doi:10.1038/s41467-022-29587-9)
Supplement: Supplementary file 1 — Supplementary Information [file 41467_2022_29587_MOESM1_ESM.pdf]

Supplementary Information for

Double–Layer Geodesic and Gradient-Index Lenses

Qiao Chen, Simon A. R. Horsley, Nelson J. G. Fonseca, Tomáš Tyc, and Oscar Quevedo–Teruel

SUPPLEMENTARY FIGURE 1

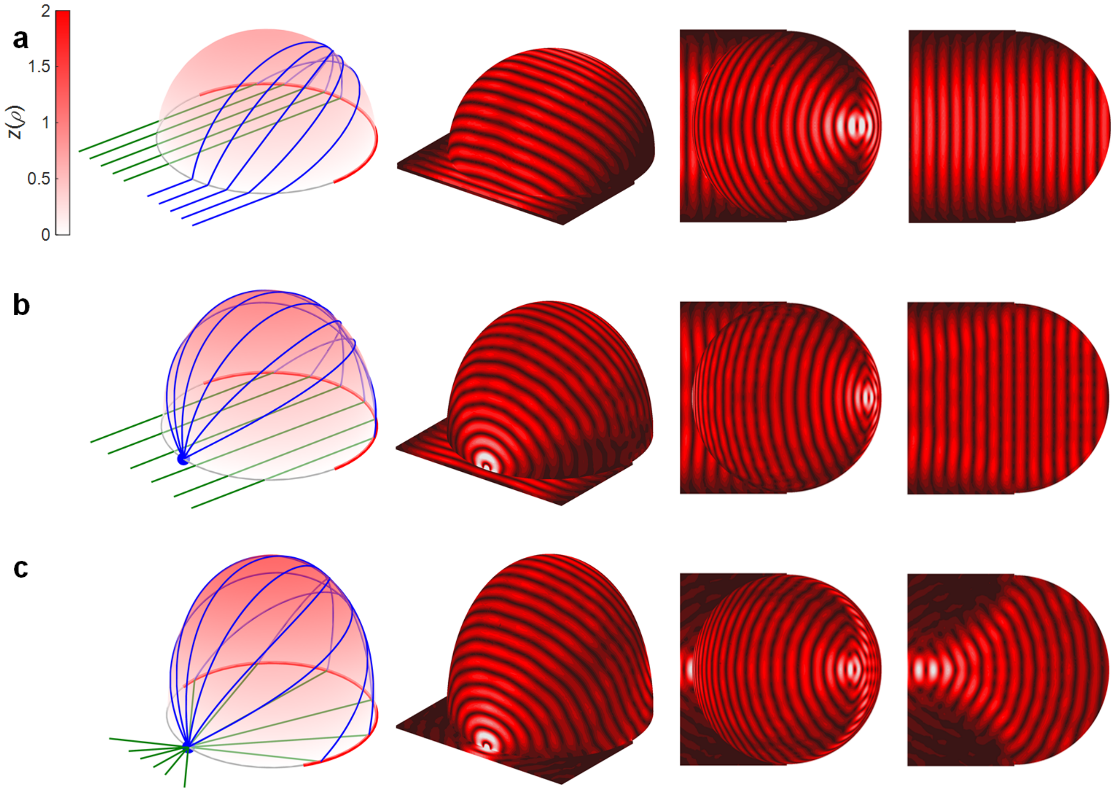

Supplementary Figure 1: *Geodesic double–layer lenses.* 3 typical examples are demonstrated with both ray-tracing and full-wave simulation (respectively, in perspective, top, and bottom views): **a.** Double–layer "invisible" lens (fed by plane wave in simulation) with $r_\mathrm{s} = \infty$, $r_\mathrm{i} = \infty$, **b.** Double–layer Luneburg lens with $r_\mathrm{s} = 1$, $r_\mathrm{i} = \infty$, **c.** Double–layer Maxwell fisheye lens with $r_\mathrm{s} = 1$, $r_\mathrm{i} = 1$.

SUPPLEMENTARY FIGURE 2

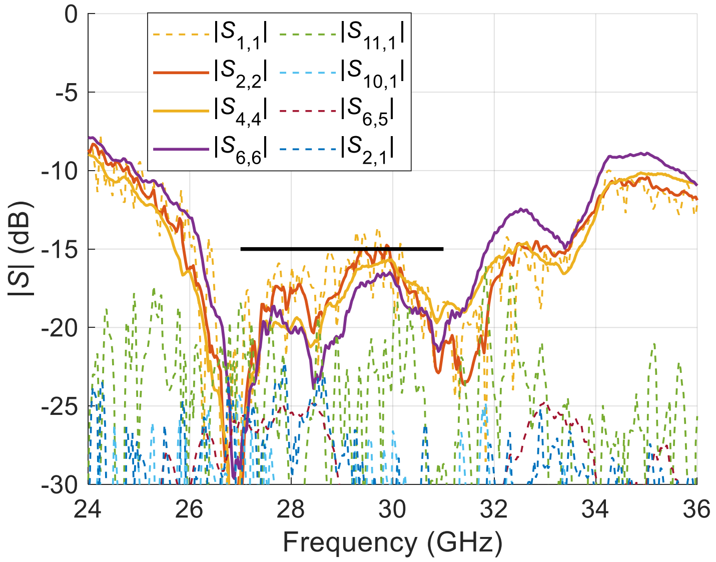

Supplementary Figure 2: *Measured scattering parameters of the geodesic double–layer lens antenna.* For brevity, only selected results are presented, and only the worst mutual-coupling coefficients are given. The coaxial-to-waveguide transition for testing works from 27 to 31 GHz.

SUPPLEMENTARY TABLE 1

Supplementary Table I: *Measured realized gain of the prototype.*

	Port 6	Port 5	Port 4	Port 3	Port 2	Port 1
26 GHz	20.4	20.4	20.6	20.5	19.8	18.0
28 GHz	20.7	20.7	20.7	20.6	20.0	18.2
30 GHz	21.1	20.8	20.9	20.7	20.0	18.5
32 GHz	21.0	21.0	21.0	21.1	20.5	19.0
